# In Vitro Antiviral Activity of Nordihydroguaiaretic Acid against SARS-CoV-2

**DOI:** 10.3390/v15051155

**Published:** 2023-05-11

**Authors:** Erendira Villalobos-Sánchez, Daniel García-Ruiz, Tanya A. Camacho-Villegas, Alejandro A. Canales-Aguirre, Abel Gutiérrez-Ortega, José E. Muñoz-Medina, Darwin E. Elizondo-Quiroga

**Affiliations:** 1 Medical and Pharmaceutical Biotechnology Unit, Center of Research and Assistance in Technology and Design of the State of Jalisco, Guadalajara 44270, Mexico; ervillalobos_al@ciatej.edu.mx (E.V.-S.);; 2 CONACYT, Medical and Pharmaceutical Biotechnology Unit, Center of Research and Assistance in Technology and Design of the State of Jalisco, Guadalajara 44270, Mexico; 3 Coordinación de Calidad de Insumos y Laboratorios Especializados, Instituto Mexicano del Seguros Social, México City 27170, Mexico

**Keywords:** SARS-CoV-2 antivirals, NDGA, *Larrea tridentata*

## Abstract

The coronavirus infectious disease 2019 (COVID-19) is caused by the severe acute respiratory syndrome coronavirus 2 (SARS-CoV-2) and has been spreading rapidly worldwide, creating a pandemic. This article describes the evaluation of the antiviral activity of nordihydroguaiaretic acid (NDGA), a molecule found in Creosote bush (*Larrea tridentata*) leaves, against SARS-CoV-2 in vitro. A 35 µM concentration of NDGA was not toxic to Vero cells and exhibited a remarkable inhibitory effect on the SARS-CoV-2 cytopathic effect, viral plaque formation, RNA replication, and expression of the SARS-CoV-2 spike glycoprotein. The 50% effective concentration for NDGA was as low as 16.97 µM. Our results show that NDGA could be a promising therapeutic candidate against SARS-CoV-2.

## 1. Introduction

Severe acute respiratory syndrome coronavirus 2 (SARS-CoV-2) is a highly transmissible virus that emerged in late 2019 and has caused a global pandemic named Coronavirus infectious disease 2019 (COVID-19) [1]. Coronaviruses (CoVs) are widespread among animal species, including bats, rodents, birds, and humans [2]. In humans, coronavirus may exhibit different clinical courses, ranging from asymptomatic infections to severe respiratory manifestations that can lead to death. The main symptoms reported include fever, chills, rigor, myalgia, malaise, diarrhea, cough, dyspnea, loss of smell or taste, and pneumonia [3]. 

CoVs are spherical viruses containing a positive-sense single-stranded RNA genome and structural proteins including the spike (S), envelope (E), membrane (M) and nucleocapsid (N), as well as several non-structural proteins [4]. Like other viruses, SARS-CoV-2 hijacks the host cell machinery and multiplies through viral attachment, fusion, penetration, uncoating, transcription, translation, and release of new virions. Viral entry is the first step in infection and one of its most important processes, in which spike glycoprotein recognizes and uses the angiotensin-converting enzyme 2 receptor (ACE2) to access host cells and then mediates membrane fusion to allow the viral genome to be released into the cytoplasm [5,6]. The ACE2 receptor is known to be present in numerous human organs (lung, heart, stomach, small intestine, colon, skin, lymph nodes, thymus, bone marrow, spleen, liver, kidney, and brain) which may help explain the main disease manifestations [7,8,9,10]. Despite some approved antiviral drugs against SARS-CoV-2, such as Remdesivir [11], Molnupiravir [12] and Nirmatrelvir [11], the development of new antiviral agents to treat hospitalized patients is urgently required. Even though current variants are less deadly, researchers have emphasized the need for new antiviral molecules because more pathogenic variants of SARS-CoV-2 could emerge [13], reaffirming the need for new molecules with antiviral activity.

In the search for new drugs, some plants have been used as an important source of molecules that could provide novel therapeutic compounds such as antivirals. Previous studies have reported compounds obtained from functional foods with antiviral activity against SARS-CoV-2, such as curcumin, quercetin, epigallocatechin gallate, thymoquinone, and sulforaphane [14,15,16]; several of these phytomolecules have a remarkable potential to act as antipathogenic agents against bacteria, fungi and viruses [17,18,19]. In this sense, therapeutic compounds with different bioactivities, such as antibacterial, antioxidant, antiapoptotic, antiparasitic, anticarcinogenic and antiviral have been isolated from the leaves of the *Larrea tridentata* plant (Creosote bush) [20,21,22,23,24,25]. The reported molecule responsible for these activities is nordihydroguaiaretic acid (NDGA) (IUPAC name: 4-[4-(3,4-dihydroxyphenyl)-2,3-dimethylbutyl] benzene-1,2-diol), also named masoprocol. NDGA is a phenolic lignan present in the resin of *L. tridentata* leaves [26]. The best characterized antiviral effects of NDGA and its derivates have been against Dengue virus (DENV), Zika virus (ZIKV), Hepatitis C virus (HCV), West Nile virus (WNV), Fort Sherman virus (FSV), Influenza virus (IV), Herpes Simplex-1 virus (HSV-1), and Human Immunodeficiency virus (HIV) [27,28,29,30,31,32]. To our knowledge there are no reports of NDGA antiviral activity against SARS-CoV-2. Thus, this work aimed to assess the in vitro antiviral activity of nordihydroguaiaretic acid, the most relevant active compound of *L. tridentata,* against SARS-CoV-2 infection.

## 2. Materials and Methods

All procedures for viral culture followed laboratory biosafety guidelines and were conducted in the CIATEJ biosafety level 3 facility. This study was approved by the Biosecurity Committee in the Centro de Asistencia en Tecnología y Diseño del Estado de Jalisco A. C. (First Ordinary session, March 2021).

### 2.1. Cells and Viruses

Vero CCL-81 cells (ATCC) were cultivated in Dulbecco’s modified Eagle’s medium (DMEM) supplemented with 10% fetal bovine serum (FBS, Gibco-Life Technologies, Carlsband, CA, USA). The SARS-CoV-2 strain (GenBank number: ON457663.1) used in the experiments was a clinical isolate provided by Hospital Civil de Guadalajara Fray Antonio Alcalde, identified by RT-PCR using the primers and probes described by the WHO for the diagnostic detection of E and RdRp genes of the SARS-CoV-2 virus. The sequenced genome was analyzed with the pathogen-tracking resource NextStrain.org, which determined that the SARS-CoV-2 strain is closely related to the pangolin lineage B.1.1. The variants of SARS-CoV-2 Alpha B.1.1.7. (GISAID ID: EPI_ISL_3556930), Delta B.1.617.2 (GISAID ID: EPI_ISL_8910780), and Omicron BA.2 (GISAID ID: EPI_ISL_9570061) were kindly provided by Dr. José Esteban Muñoz from Coordinación de Calidad de Insumos y Laboratorios Especializados, Instituto Mexicano del Seguro Social.

### 2.2. Antiviral Molecule

NDGA powder was purchased from Sigma Aldrich (St. Louis, MI, USA). The purity of NDGA was ≥90% (HPLC). The powder was dissolved in 1 mL of sterile dimethyl sulfoxide (DMSO) to obtain a 0.01 M stock solution, sterilized by filtration, and further working dilutions were made from the stock in DMEM, 1% FBS.

### 2.3. Virus Titration

In order to determine the number of infectious viral particles in the suspension, SARS-CoV-2 titration was performed by a 50% tissue culture infectious dose assay (TCID_50_). Vero cells were seeded in 96-well plates, prepared with DMEM containing 10% FBS. The plate was incubated for 24 h at 37 °C with 5% CO_2_ to obtain a confluent monolayer. The medium from the plate was removed and ten-fold serial dilutions of the virus (100 µL/well) in DMEM, 1% FBS were added to the cell monolayers. The plate was incubated for 72 h at 37 °C with 5% CO_2_. Subsequently, the number of wells that were positive for cytopathic effect (CPE) for each dilution was counted. A Reed and Muench calculation (Reed and Muench, 1938) was then performed to determine the 50% infectious dose (TCID_50_/mL) [33].

### 2.4. Maximum Non-Cytotoxic Dose

To find the NDGA concentration to use in the antiviral assays, the toxicity of the compound was evaluated in Vero cells looking for the maximum non-cytotoxic dose (MNCD), by observation of changes in cell morphology under an inverted microscope. We used a precultured 96-well plate, with a concentration of 300,000 Vero cells/mL incubated for 24 h at 37 °C with 5% CO_2_. Then, the medium was discarded and replaced with 100 µL/well of NDGA at different concentrations (15, 20, 35, 50, 100, 150 and 200 µM). The plate was then incubated for 48 h at 37 °C and 5% CO_2_. Control Vero cells were incubated only with DMEM, 1% FBS, and 0.01% DMSO. Two independent experiments were performed with eight replicates for each concentration level (*n* = 8). Cytotoxicity was determined by loss of monolayer integrity, granulation, and vacuolization. 

### 2.5. Cell Viability

To estimate the cell viability of the MNCD in the previous assay, the MTT methodology was applied. Cell viability of NDGA was assessed on mitochondrial reduction of 3-[4,5-dimethylthiazol-2-yl]–2,5-diphenyltetrasodium bromide (MTT) (Roche, Mannheim, Germany), to a purple formazan reaction product by living cells. We used the 96-well plate where the MNCD was previously evaluated. Then, 10 µL of MTT solution (5 mg/mL) was added to each well and the plate was incubated for 2 h at 37 °C. Two independent experiments were performed with four replicates for each concentration level (*n* = 4). After incubation, the crystals formed were solubilized by adding 100 µL of isopropanol to each well, and the absorbance at 570 nm was measured by using the xMark™ microplate Spectrophotometer (Bio-Rad Laboratories, Hercules, CA, USA). The cell viability rate was calculated according to the following equation: percentage of cell viability = (A/B) × 100%, where “A” is the absorbance of cells treated with NDGA, and “B” is absorbance of the untreated cells. The 50% cytotoxicity concentration (CC_50_) was defined as the concentration of NDGA that reduced the cell viability of treated cells by 50% when compared to control cells. CC_50_ was calculated from the mean dose–response curve of two separate experiments by non-linear regression analysis.

### 2.6. Antiviral Assay

To determine the possible antiviral activity of NDGA against SARS-CoV-2, we challenged the MNCD selected against 100 TCID_50_/mL of SARS-CoV-2 in Vero cells, looking for the CPE inhibition of the viral infection. Vero cells were seeded to obtain confluent monolayers using a 96-well plate. Then, the plate was emptied and washed with phosphate-buffered saline (PBS), and 100 µL of the mix was deposited per well. DMSO was included as a control and used at 0.01% final concentration. The plate was incubated at 37 °C in an atmosphere of 5% CO_2_ for 96 h. Cell cultures were inspected daily under the inverted microscope for detectable alterations. Two independent experiments were performed with eight replicates for each concentration level (*n* = 8). After 96 h post-infection, supernatants were collected and frozen at −80 °C for further RNA extraction and viral genome quantification by real-time qPCR. The 50% effective concentration (EC_50_) and 90% effective concentration (EC_90_) were calculated from the mean dose–response curve by non-linear regression analysis. The results were expressed using the selectivity index (SI = CC_50_/EC_50_). Images were captured with an Optikam WiFi-4083 camera. 

### 2.7. Plaque Reduction Assay

We performed a viral plaque reduction assay to evaluate the inhibitory action of NDGA against SARS-CoV-2 infection in Vero cells. Precultured 24 well plates were seeded to achieve monolayer formation. The plates were emptied and washed twice with 1X PBS. Then, 100 TCID_50_/mL of SARS-CoV-2 B.1.1 was mixed with MNCD of NDGA in DMEM—1% FBS and 200 µL of the mixture was added to each well in quadruplicate. The plate was incubated for viral absorption for 1 h at 37 °C. Later, the inoculum was removed and replaced with 1 mL of overlay medium (1:1 dilution of 3% carboxymethyl cellulose in 2X DMEM with 2% FBS). The plate was incubated for 96 h in an atmosphere of 5% CO_2_ at 37 °C. Afterward, the overlay medium was removed by decanting, followed by three PBS washes to remove the excess of the semi-solid medium. Plaques were stained and fixed with 1% crystal violet in methanol at room temperature for 15 min. Subsequently, the stain solution was removed, and monolayers were washed with tap water until plaques were revealed. 

### 2.8. Time-of-Drug Addition Assays

The time-of-addition assays were performed to evaluate whether NDGA’s antiviral activity varied during different viral infection stages of SARS-CoV-2 using three different approaches: simultaneous, pre-infected, and virucidal. In all assays, we used 24-well plates to test the ability of the MNCD of NDGA to reduce plaque formation using 250 TCID_50_/well of the SARS-CoV-2 B.1.1 virus. 

#### 2.8.1. Simultaneous Assay

For viral entry evaluation, the MNCD of NDGA was mixed with 250 TCID_50_ of SARS-CoV-2 in a 1.5 mL tube and added onto the Vero cell monolayer for 1 h at 37 °C. After, the medium was discarded and the cell monolayer was washed with PBS to remove unattached viruses, 1 mL/well of overlay medium was added and the plate was incubated for 4 days at 37 °C.

#### 2.8.2. Pre-Infection Assay

For the post-virus internalization evaluation, Vero cell monolayers were infected with 250 TCID_50_/well for 1 h at 37 °C. Then, MNCD of NDGA was added to cells for 1 h and incubated again for an additional hour at 37 °C. Subsequently, cell monolayers were washed with 1X PBS to remove unattached viruses and 1 mL/well of overlay medium was added; the plate was incubated for 4 days at 37 °C.

#### 2.8.3. Virucidal Assay

For the virucidal evaluation, MNCD of NDGA was directly incubated with 250 TCID_50_ of SARS-CoV-2 at room temperature in 1.5 mL microtube for 1 h. Afterwards, the mixtures were added to the Vero cell monolayers and incubated at 37 °C for 1 h. Then, cell monolayers were washed with 1X PBS to remove unattached viruses and 1 mL/well of overlay medium was added; the plate was incubated for 4 days at 37 °C.

### 2.9. Protein Extraction and Western Blot Analysis

Immunoblot analysis was carried out to evaluate the accumulation of viral proteins in treated and SARS-CoV-2 B.1.1 infected Vero cells. Briefly, the cells were washed with 1X PBS and lysed with 50 µL/well ice-cold lysis buffer (RIPA lysis buffer). The protein concentration was determined using the Bradford method (BioRad Laboratories Inc., Hercules, CA, USA).

Protein samples (30 µL) were boiled for 5 min and separated by SDS-PAGE on a 12% acrylamide gel at 100 V for 1 h. Afterwards, the gel was transferred to a nitrocellulose membrane (0.45 µm) (BioRad Laboratories Inc., Hercules, CA, USA). The membrane was blocked with blocking buffer (PBST; 0.05% tween 20 in saline phosphate buffer containing 5% low-fat milk Svelty^®^) for 1 h under constant orbital stirring. Afterwards, the membrane was incubated with orbital stirring overnight at 4 °C with primary antibodies, rinsed, and incubated with orbital stirring with the corresponding HRP-conjugated secondary antibodies for 120 min. Membranes were washed two times, and finally, protein bands were revealed using tetramethyl benzidine (TMB) (BioRad Laboratories Inc., Hercules, CA, USA). 

The SARS-CoV-2 spike protein (RBD) monoclonal antibody (HL257) (Thermo Fisher Scientific, Walthman, MA, USA; MA5-36253, Rabbit) was diluted 1:5000 in blocking buffer containing 2.5% low-fat milk and detected with HRP-conjugated anti-rabbit IgG antibody, diluted 1:20,000 (Vector laboratories, Burlingame, CA, USA; PI-1000). β-actin antibody was used as constitutively expressed protein (Santa Cruz Biotechnology, Dallas Texas, USA; sc-47778), diluted at 1:10,000 and detected with HRP-conjugated anti-mouse-IgG antibody (Santa Cruz Biotechnology, Santa Cruz, CA, USA).

### 2.10. Viral Genome Quantification

To quantify the viral genomic copies of SARS-CoV-2 after the NDGA treatment, compared to untreated Vero cells (positive control). Viral RNA was isolated from cell culture supernatants in triplicate using a QIAamp Viral RNA kit (QiagenTM, Hilden, Germany). A total of 100 µL was processed according to the manufacturer’s instructions. Viral RNA was resuspended in 60 µL elution buffer. RNAs were kept on ice and qPCR was carried out immediately after RNA extraction, according to the Instituto de Diagnóstico y Referencia Epidemiológicos Dr. Manuel Martínez Báez (InDRE) guidelines, using the primers and probes described by the WHO for the diagnostic detection of SARS-CoV-2; (forward E_Sarbeco_F1 5′ ACAGGTACGTTAATAGTTAATAGCGT 3′, reverse E_Sarbeco_R2 5′ ATATTGCAGCAGTACGCACACA 3′ and probe E_Sarbeco_P1 5′ FAM-ACACTAGCCATCCTTACTGCGCTTCG-BHQ1 3′). This assay amplifies a 113 nt fragment of the virus E gene. Reactions were carried out in a CFX96 Real-Time thermocycler (Bio-Rad) using a Super ScriptTM III PlatinumTM One-Step qRT-PCR System kit (Invitrogen). A standard curve with four triplicate dilutions was generated, using a plasmid containing SARS-CoV-2 genome fragments recognized by the qPCR probe provided by Instituto de Biotecnología de la Universidad Autónoma de México (IBT, UNAM).

### 2.11. Statistical Analysis

All data were analyzed with GraphPad Prism 8.0.2. Data were presented as mean ± SEM. Statistical differences were evaluated by Student’s *t*-test based on Shapiro–Wilk normality test. A *p*-value ≤ 0.05 was considered significant, with **** *p* ≤ 0.001. 

## 3. Results

### 3.1. Cytotoxicity Assay to Identify the NDGA Maximum Non-Cytotoxic Dose

**Figure 1:**
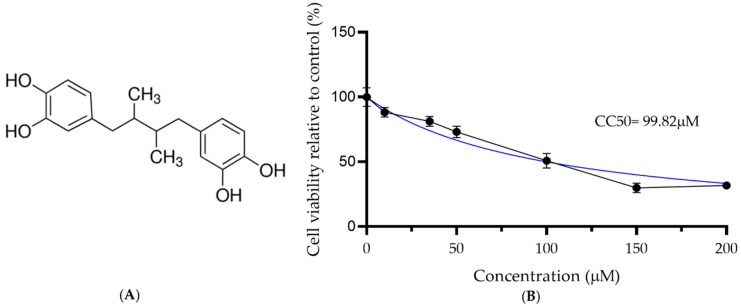
Cytotoxicity of nordihydroguaiaretic acid in Vero cells. (**A**) Chemical structure of NDGA; (**B**) Cell viability of Vero cells treated with NDGA (≥90% purity) after 48 h treatment, using the MTT assay. Each value represents the mean of two experiments with four replicates ± standard deviation (SD). CC_50_ value was determined using non-linear regression with GraphPad Prism 8, and the blue line represents the tendency line of analysis.

To identify the MNCD, NDGA concentrations of 15, 20, 35, 50, 150 and 200 µM were tested. Concentrations of 35 and 50 µM did not show damage to the cell monolayer, while higher concentrations showed morphological changes, such as rounding and detachment. Therefore, the 35 and 50 µM concentrations were selected to be used in the antiviral activity tests against SARS-CoV-2. Cell viability was also evaluated by the MTT assay. Cell viability was 88.1, 81.2, 73.04, 50.77, 29.87, and 31.64% for concentrations of 10, 35, 50, 100, 150 and 200 µM, respectively (Figure 1). The CC50 was 99.82 µM ± 2.23, as shown in Table 1. 

### 3.2. Antiviral Activity of NDGA

**Figure 2:**
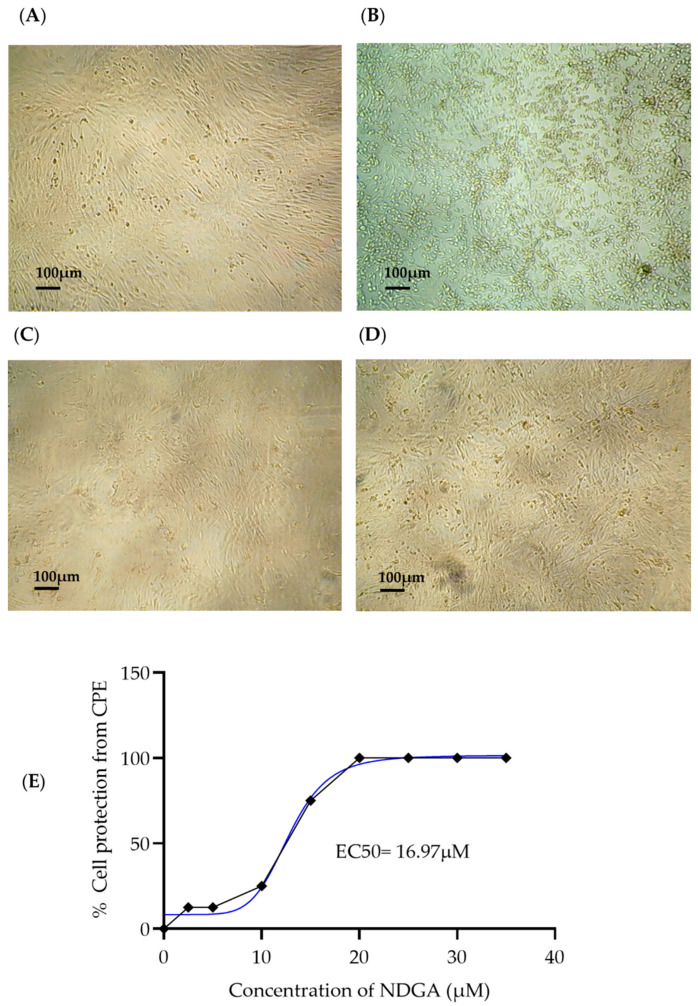
Inverted microscopic representative photographs of cell cultures at 96 h post-infection. Vero CCL-81 cells were infected with 100 TCID_50_/mL of SARS-CoV-2 and treated with two NDGA concentrations. (**A**) Negative control (untreated Vero cells); (**B**) SARS-CoV-2 positive control presenting CPE (black arrow: cell rounding; yellow arrow: detachment); (**C**) Infected Vero cells treated with NDGA 35 µM; (**D**) Infected Vero cells treated with NDGA 50 µM. (**E**) Dose–response curve analyses were performed against 100 TCID_50_ viral concentrations. Cytopathic effect reduction was expressed as the percent protection from CPE (no CPE appearance in replicates *n* = 8) in two experiments. The EC50 value was determined using non-linear regression with GraphPad Prism 8, and the blue line represents the tendency line of analysis.

To determine the antiviral activity of NDGA, 35 and 50 µM concentrations were selected; the assay was carried out using 100 TCID_50_/mL of SARS-CoV-2 B.1.1 for 96 h. Our results showed that both NDGA concentrations used in this experiment avoided viral infection since no CPE caused by SARS-CoV-2 appeared in any of the replicates; in contrast, all replicates in the positive control (Vero cells inoculated with 100 TCID_50_/mL SARS-CoV-2) developed CPE (cells rounded and detached from the monolayer) (Figure 2A–D). On the other hand, we also evaluated the antiviral activity of 35 µM NDGA against the SARS-CoV-2 variants of interest, Alpha, Delta, and Omicron, where no CPE appearance was observed for any of their replicates (*n* = 8). 

To determine NDGA EC_50_ and EC_90_, ten different concentrations between the 2.5–35 µM range were evaluated for their ability to reduce the virus-induced CPE. Our results showed in vitro antiviral activity against SARS-CoV-2 B.1.1, with an estimated EC_50_ value of 16.97 µM and EC_90_ of 25.64 µM. The selectivity index (SI) was determined by quantifying the relationship between CC_50_ and EC_50_. The EC_50_ and EC_90_ were determined with GraphPad Prism 8 (GraphPad Software, San Diego, CA, USA) using non-linear regression. The SI of NDGA was 5.88, as shown in Table 1. 

### 3.3. NDGA Mainly Acts at Early Stages of SARS-CoV-2 Infection

**Figure 3:**
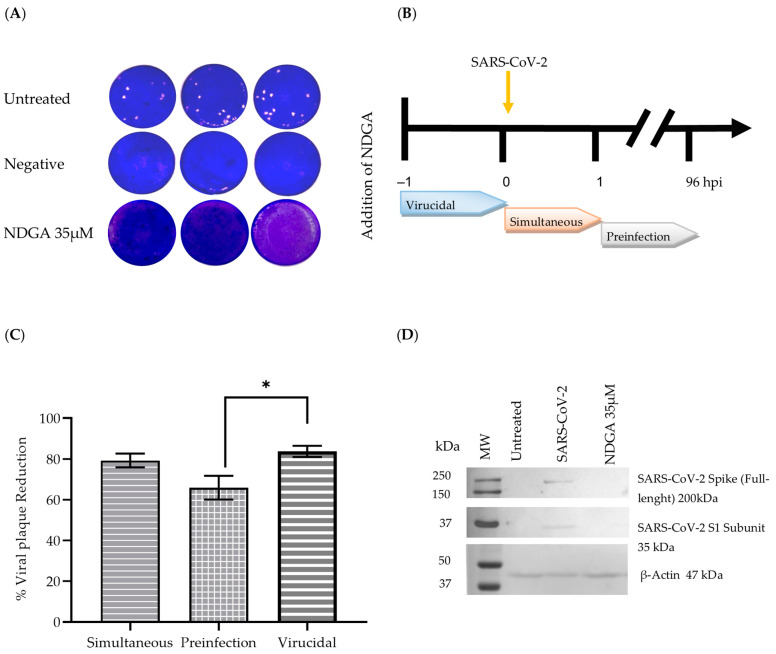
Antiviral activity of NDGA against SARS-CoV-2 by viral plaque reduction assay at 96 h post-infection. (**A**) Simultaneous infection assay with 35 µM NDGA, plus 100 TCID_50_/mL of SARS-CoV-2; (**B**) Diagram of the time of drug assay; (**C**) Time of addition analysis of NDGA against 250 TCID_50_/mL of SARS-CoV-2 infection. The experiments were carried out in quadruplicate, and the percentage of reduction is represented as the mean ± S.D. * *p* ≤ 0.05, as compared with control; (**D**) Western blot analysis. SARS-CoV-2 spike protein was detected in the positive control but not with 35 µM of NDGA treatment. Bio-Rad Precision Plus Standards were loaded in line 1.

The antiviral activity of NDGA against SARS-CoV-2 was also determined by measurement of viral plaque formation (plaque reduction assay) using the same conditions described in 3.2. No viral plaques were observed in the treatment, suggesting that 35 µM of NDGA can inhibit viral plaque formation after SARS-CoV-2 infection (Figure 3A).

To determine the mechanism of action of NDGA, time-of-drug addition assays were performed to indicate the time at which the compound reaches the maximum antiviral activity by measuring the reduction in the number of viral foci (Figure 3B). Since CPE using 100 TCID_50_/mL of the virus was completely inhibited by 35 µM NDGA, we hypothesized that the “time-of-addition assays” would not show differences, so we decided to increase the viral concentration to 250 TCID_50_/mL for these tests. In the simultaneous assay, 250 TCID_50_/mL plus 35 µM NDGA were added onto Vero cells for 1 h to allow absorption simultaneously. The treatment reduced viral plaque by approximately 79.31% compared to the untreated control. A similar result was seen when NDGA was incubated for 1 h before viral infection (virucidal effect) with a plaque reduction of 83.78%. In contrast, when NDGA was added after 1 h of viral infection (pre-infection), the viral plaque reduction was 65.97% (Figure 3C). 

### 3.4. Western Blot Analysis

Cell lysates were analyzed for expression of viral proteins on Western blotting, using the SARS-CoV-2 Spike protein (RBD) antibody. The results confirmed the antiviral effect of NDGA on the expression of SARS-CoV-2 spike protein. The full-length protein was readily identified in the positive control, while in the negative control (non-infected Vero cells) and treatment (Vero cells infected with 100 TCID_50_/mL of SARS-CoV-2 B.1.1 variant and treated with NDGA 35 µM) the protein was not detected (Figure 3D).

### 3.5. Inhibitory Effect of NDGA against Viral Replication of SARS-CoV-2 Variants

**Figure 4:**
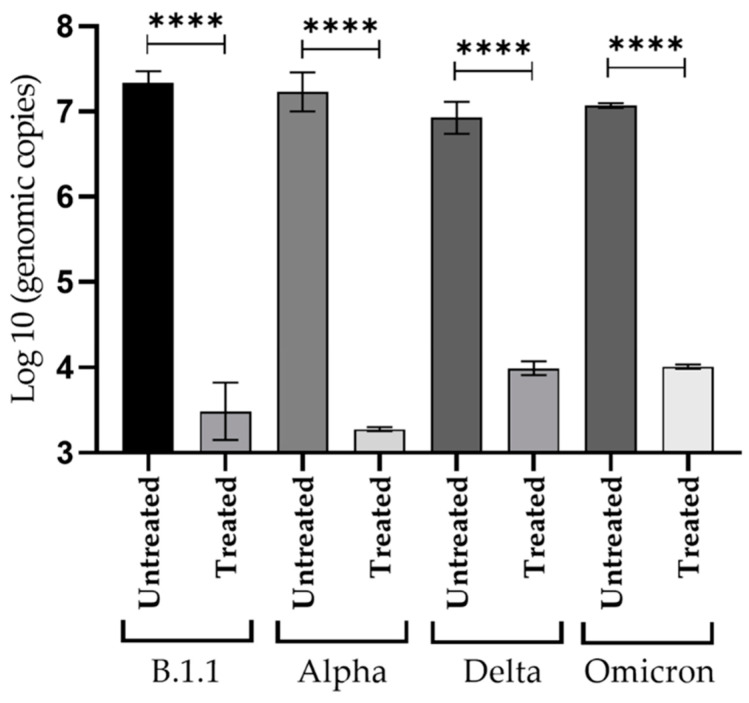
Quantification of viral genome replication in cell culture. Vero cells were treated with NDGA 35 µM and infected simultaneously with 100 TCID_50_/mL of each SARS-CoV-2 variant, for 96 h. as described in Section 2.6. All assays were performed in triplicate. Viral RNA from positive control (untreated) supernatants or treated cultures were measured by real-time RT-qPCR. The student *t*-test was performed, and data is reported as mean values ± SD (**** *p* < 0.001).

The number of extracellular viral genome copies was determined by quantitative RT-qPCR in cell culture supernatants where CPE inhibition was tested. The supernatant collection was performed in triplicate, taking 100 µL of the cell medium at 96 h post-infection. RT-qPCR of supernatants recovered from cells infected with 100 TCID_50_/mL and treated with 35 µM of NDGA, showed a significant reduction in genome copy number with respect to the positive control. The highest difference was observed for the Alpha variant, where the reduction was as high as 3.95 log^10^, followed by 3.85, 3.05 and 2.93 for B.1.1., Omicron, and Delta variants, respectively (Figure 4). The differences were statistically significant, with *p*-value < 0.001 in treatments compared with their respective positive control. 

## 4. Discussion

SARS-CoV-2 remains a potential threat to public health, not only because the virus constantly mutates reducing vaccine protective efficacy, but also because it could re-emerge with more pathogenic variants, or new β-coronaviruses zoonoses could appear de novo [13]. Among the large number of detected SARS-CoV-2 variants, some of these can pose a public health risk, as they are more contagious, however, all strains represent a risk of infection [34]. 

Antiviral research during the SARS-CoV-2 pandemic resulted in the identification and use of some molecules that may target viral replication directly. For example, Paxlovid^®^ (a blend of nirmatrelvir with ritonavir), where the nirmatrelvir acts as an inhibitor of the main protease MPro, an essential protein for viral replication and highly conserved in beta coronaviruses [35,36]. Other FDA-approved antiviral compounds are remdesivir and molnupiravir. Both are nucleoside analogs that act by inhibiting the viral enzyme RdRp [37]. Nevertheless, there are very few antiviral treatments for the infection caused by SARS-CoV-2. Due to its ability to inhibit viral replication of other viruses, we decided to evaluate the anti-SARS-CoV-2 effect of NDGA using Vero cell cultures.

Our findings show that no significant in vitro toxicity was observed with NDGA at 35 µM concentration in Vero cells. Similar to our results, previous studies reported low in vitro toxicity in mammalian cells. Martinez et al. reported a maximal non-cytotoxic concentration of 90.4 µM in LLC-MK2 cells [28]; Soto-Acosta et al., found that 100 µM of NDGA did not reduced cell viability in Vero and U937-DC SIGN cells [29]; Koob et al., reported that NDGA was cytotoxic to tendon fibroblast cells at concentrations above 100 μM [38]. Finally, the results of Merino Ramos et al. showed that 35 µM of NDGA showed cytotoxicity above 80% by measuring the cellular ATP content with the Cell Titer-Glo luminescent cell viability test, while we performed an MTT assay and found cell viability of 86% at the same concentration [27].

The antiviral activity of NDGA was previously reported on ZIKV, DENV, WNV, Sherman virus, and influenza virus; however, to our knowledge, the antiviral effect on SARS-CoV-2 virus had not been addressed before. Our study found that NDGA has antiviral activity against SARS-CoV-2 in vitro. First, we explored the infectivity of SARS-CoV-2 in Vero cells to identify the minimal viral concentration that can form CPE in all replicates, and we found that 100 TCID_50_/mL is an adequate concentration to perform our antiviral assays [39,40,41]. Surprisingly, the results of antiviral assays against 100 TCID_50_/mL showed a complete SARS-CoV-2 CPE inhibition using 35 µM NDGA, compared to the positive control treated with 0.01M of DMSO for all variants tested. To confirm our results, we performed a viral plaque reduction assay using the same conditions and found no plaques on the Vero cell monolayers.

The time-of-drug-addition assay can provide a preliminary understanding of the antiviral activity. The simultaneous assay can provide information about viral attachment to cell receptors to initiate cell entry. In contrast, the pre-infection assay could explain the antiviral effect during the post-entry steps, such as genome translation and replication, virus assembly, and viral release from the cells [42]. Our results revealed that NDGA acts mainly in the early stages of SARS-CoV-2 infection and after viral adsorption. It has been reported that NDGA could inhibit the viral infection of flaviviruses by different mechanisms. Several studies indicate that NDGA inhibits genome replication and viral assembly of DENV, ZIKV, HCV and WNV [27,29]. In addition, NDGA has also been found to be a potent regulator of the sterol regulatory element-binding proteins (SREBP) pathway [43]. As with these flaviviruses, some recent findings have shown dependence on lipid metabolism during SARS-CoV-2 infection, to enhance the expression of key enzymes of the SREBP pathway to invade cells and replicate [44,45]. Additionally, SARS-CoV-2 appropriates lipid droplets for viral membrane formation and energy production [45]. Taken together, these results could explain two possible antiviral mechanisms of NDGA, since no CPE was observed when cells were treated in the early stages of infection and could be explained as inhibition of viral entry or replication. Another possible mechanism could be related to lipid regulation during post-infection experiments since both antiviral mechanisms could be involved during the cell treatment with NDGA but further analyses should be performed to understand the correct route.

In our experimental conditions, 35 µM of NDGA inhibits SARS-CoV-2 infection with an EC_50_ = 16.97 µM, CC_50_ 99.82 and SI = 5.88. The EC_50_ dose of NDGA found was lower in contrast with other drugs such as rivabirin (EC_50_ = 109.50 μM, CC_50_ > 400 μM, SI > 3.65), penciclovir (EC_50_ = 95.96 μM, CC_50_ > 400 μM, SI > 4.17) and favipiravir (EC_50_ = 61.88 μM, CC_50_ > 400 μM, SI > 6.46), however, other molecules that have been used for the disease treatment such as remdesivir (EC_50_ = 0.77 μM; CC_50_ > 100 μM; SI > 129.87) and chloroquine (EC_50_ = 1.13 μM; CC_50_ > 100 μM, SI > 88.50) have lower EC_50_ concentrations in contrast to NDGA [46].

Given that many viruses can potentially develop drug resistance such as nirma-trelvir resistant SARS-CoV-2 variants, it could be suggested that combinations of approved antivirals in synergy with NDGA can be used to decrease viral load as other researchers have concluded [47,48]. Furthermore, regarding the properties of NDGA in the SREBP pathway [29], the enzyme regulation could be directly involved in antiviral activity, which could suggest that the treatment with NDGA might be useful for the development of a potent antiviral agent to prevent viral dissemination along tissues and to regulate the lipogenesis induced by SARS-CoV-2.

Our results revealed that NDGA has potent antiviral activities against SARS-CoV-2 in Vero cells and represents a promising approach to recognize it as an antiviral molecule to treat different viral illnesses. In summary, no evidence of viral replication was found using NDGA at a final concentration of 35 µM against 100 TCID_50_/mL of the four variants of interest used in this study. Under the inverted microscope, no appearance of CPE was observed. When the viral load was quantified in these cultures using RT-PCR, no genomic copy increase was detected in the supernatants. Likewise, when looking for the spike protein by Western blot, it was absent in the treatments, and PRNT counted no plaques.

Finally, the results of this study explored the antiviral activity of NDGA against in vitro infection of SARS-CoV-2 and they point out that this molecule could have the potential to be used as a therapeutic agent against COVID-19, although further studies to identify the specific action mechanisms are required. Furthermore, developing NDGA derivatives with improved specificity promises to identify antiviral agents not only against SARS-CoV-2, but also against other RNA viruses.

## Figures and Tables

**Table 1:** Inhibitory effects of NDGA on SARS-CoV-2 in vitro.

Compound	CC_50_	EC_50_	EC_90_	SI
NDGA	99.82	16.97	25.64	5.88

CC_50_, 50% cytotoxic concentration; EC_50_, 50% effective concentration; EC_90_, 90% effective concentration; SI, Selectivity index.

## Data Availability

Not applicable.

## References

[1] Zhu N., Zhang D., Wang W., Li X., Yang B., Song J., Zhao X., Huang B., Shi W., Lu R. (2020). A novel coronavirus from patients with pneumonia in China, 2019. N. Engl. J. Med..

[2] Ye Z.-W., Yuan S., Yuen K.-S., Fung S.-Y., Chan C.-P., Jin D.-Y. (2020). Zoonotic origins of human coronaviruses. Int. J. Biol. Sci..

[3] Çalıca Utku A., Budak G., Karabay O., Güçlü E., Okan H.D., Vatan A. (2020). Main symptoms in patients presenting in the COVID-19 period. Scott. Med. J..

[4] Malik Y.A. (2020). Properties of coronavirus and SARS-CoV-2. Malays. J. Pathol..

[5] Jackson C.B., Farzan M., Chen B., Choe H. (2022). Mechanisms of SARS-CoV-2 entry into cells. Nat. Rev. Mol. Cell Biol..

[6] Shang J., Wan Y., Luo C., Ye G., Geng Q., Auerbach A., Li F. (2020). Cell entry mechanisms of SARS-CoV-2. Proc. Natl. Acad. Sci. USA.

[7] Weng L.-M., Su X., Wang X.-Q. (2021). Pain symptoms in patients with coronavirus disease (COVID-19): A literature review. J. Pain Res..

[8] Ftiha F., Shalom M., Jradeh H. (2020). Neurological symptoms due to Coronavirus disease 2019. Neurol. Int..

[9] Ramachandran P., Onukogu I., Ghanta S., Gajendran M., Perisetti A., Goyal H., Aggarwal A. (2020). Gastrointestinal symptoms and outcomes in hospitalized coronavirus disease 2019 patients. Dig. Dis..

[10] Sungnak W., Huang N., Bécavin C., Berg M., Queen R., Litvinukova M., Talavera-López C., Maatz H., Reichart D., Sampaziotis F. (2020). SARS-CoV-2 entry factors are highly expressed in nasal epithelial cells together with innate immune genes. Nat. Med..

[11] Lamb Y.N. (2020). Remdesivir: First approval. Drugs.

[12] Syed Y.Y. (2022). Molnupiravir: First approval. Drugs.

[13] Vicidomini C., Roviello G.N. (2023). Potential Anti-SARS-CoV-2 Molecular Strategies. Molecules.

[14] Ricci A., Roviello G.N. (2023). Exploring the Protective Effect of Food Drugs against Viral Diseases: Interaction of Functional Food Ingredients and SARS-CoV-2, Influenza Virus, and HSV. Life.

[15] Behl T., Rocchetti G., Chadha S., Zengin G., Bungau S., Kumar A., Mehta V., Uddin M.S., Khullar G., Setia D. (2021). Phytochemicals from plant foods as potential source of antiviral agents: An overview. Pharmaceuticals.

[16] Garcia-Ruiz D., Villalobos-Sánchez E., Alam-Escamilla D., Elizondo-Quiroga D. (2022). In vitro inhibition of SARS-CoV-2 Infection by dry algae powders. Sci. Rep..

[17] Savoia D. (2012). Plant-derived antimicrobial compounds: Alternatives to antibiotics. Future Microbiol..

[18] Mishra K., Sharma N., Diwaker D., Ganju L., Singh S. (2013). Plant derived antivirals: A potential source of drug development. J. Virol. Antivir. Res..

[19] Arif T., Bhosale J., Kumar N., Mandal T., Bendre R., Lavekar G., Dabur R. (2009). Natural products–antifungal agents derived from plants. J. Asian Nat. Prod. Res..

[20] Bashyal B., Li L., Bains T., Debnath A., LaBarbera D.V. (2017). Larrea tridentata: A novel source for anti-parasitic agents active against *Entamoeba histolytica*, *Giardia lamblia* and *Naegleria fowleri*. PLoS Negl. Trop. Dis..

[21] Morales-Ubaldo A.L., Rivero-Perez N., Avila-Ramos F., Aquino-Torres E., Prieto-Méndez J., Hetta H.F., El-Saber Batiha G., Zaragoza-Bastida A. (2021). Bactericidal activity of Larrea tridentata hydroalcoholic extract against phytopathogenic bacteria. Agronomy.

[22] Skouta R., Morán-Santibañez K., Valenzuela C.A., Vasquez A.H., Fenelon K. (2018). Assessing the antioxidant properties of Larrea tridentata extract as a potential molecular therapy against oxidative stress. Molecules.

[23] Morán-Santibañez K., Vasquez A.H., Varela-Ramirez A., Henderson V., Sweeney J., Odero-Marah V., Fenelon K., Skouta R. (2019). Larrea tridentata extract mitigates oxidative stress-induced cytotoxicity in human neuroblastoma SH-SY5Y cells. Antioxidants.

[24] Manda G., Rojo A.I., Martínez-Klimova E., Pedraza-Chaverri J., Cuadrado A. (2020). Nordihydroguaiaretic acid: From herbal medicine to clinical development for cancer and chronic diseases. Front. Pharmacol..

[25] Saldívar R.H.L. (2003). Estado actual del conocimiento sobre las propiedades biocidas de la gobernadora [*Larrea tridentata* (DC) Coville]. Rev. Mex. Fitopatol..

[26] Hyder P.W., Fredrickson E., Estell R.E., Tellez M., Gibbens R.P. (2002). Distribution and concentration of total phenolics, condensed tannins, and nordihydroguaiaretic acid (NDGA) in creosotebush (*Larrea tridentata*). Biochem. Syst. Ecol..

[27] Merino-Ramos T., Jiménez de Oya N., Saiz J.-C., Martín-Acebes M.A. (2017). Antiviral activity of nordihydroguaiaretic acid and its derivative tetra-O-methyl nordihydroguaiaretic acid against West Nile virus and Zika virus. Antimicrob. Agents Chemother..

[28] Martinez F., Mugas M.L., Aguilar J.J., Marioni J., Contigiani M.S., Montoya S.C.N., Konigheim B.S. (2021). First report of antiviral activity of nordihydroguaiaretic acid against Fort Sherman virus (Orthobunyavirus). Antivir. Res..

[29] Soto-Acosta R., Bautista-Carbajal P., Syed G.H., Siddiqui A., Del Angel R.M. (2014). Nordihydroguaiaretic acid (NDGA) inhibits replication and viral morphogenesis of dengue virus. Antivir. Res..

[30] Uchide N., Ohyama K., Bessho T., Toyoda H. (2005). Inhibition of influenza-virus-induced apoptosis in chorion cells of human fetal membranes by nordihydroguaiaretic Acid. Intervirology.

[31] Hwu J.R., Tseng W.N., Gnabre J., Giza P., Huang R.C.C. (1998). Antiviral activities of methylated nordihydroguaiaretic acids. 1. Synthesis, structure identification, and inhibition of tat-regulated HIV transactivation. J. Med. Chem..

[32] Park R. (2003). Inhibition of the Herpes Simplex Virus Type I by Three NDGA Derivatives: Mal.4, M_4_N, and G_4_N. Ph.D. Thesis.

[33] Ramakrishnan M.A. (2016). Determination of 50% endpoint titer using a simple formula. World J. Virol..

[34] Janik E., Niemcewicz M., Podogrocki M., Majsterek I., Bijak M. (2021). The emerging concern and interest SARS-CoV-2 variants. Pathogens.

[35] Melo-Filho C.C., Bobrowski T., Martin H.-J., Sessions Z., Popov K.I., Moorman N.J., Baric R.S., Muratov E.N., Tropsha A. (2022). Conserved coronavirus proteins as targets of broad-spectrum antivirals. Antivir. Res..

[36] Zhong W., Jiang X., Yang X., Feng T., Duan Z., Wang W., Sun Z., Chen L., Nie X., Zhu C. (2022). The efficacy of paxlovid in elderly patients infected with SARS-CoV-2 omicron variants: Results of a non-randomized clinical trial. Front. Med..

[37] Hosseini E.S., Kashani N.R., Nikzad H., Azadbakht J., Bafrani H.H., Kashani H.H. (2020). The novel coronavirus Disease-2019 (COVID-19): Mechanism of action, detection and recent therapeutic strategies. Virology.

[38] Koob T.J., Willis T.A., Hernandez D.J. (2001). Biocompatibility of NDGA‐polymerized collagen fibers. I. Evaluation of cytotoxicity with tendon fibroblasts in vitro. J. Biomed. Mater. Res..

[39] Unal M.A., Bitirim C.V., Summak G.Y., Bereketoglu S., Cevher Zeytin I., Besbinar O., Gurcan C., Aydos D., Goksoy E., Kocakaya E. (2021). Ribavirin shows antiviral activity against SARS-CoV-2 and downregulates the activity of TMPRSS2 and the expression of ACE2 in vitro. Can. J. Physiol. Pharmacol..

[40] Signer J., Jonsdottir H.R., Albrich W.C., Strasser M., Züst R., Ryter S., Ackermann-Gäumann R., Lenz N., Siegrist D., Suter A. (2020). In vitro antiviral activity of Echinaforce^®^, an Echinacea purpurea preparation, against common cold coronavirus 229E and highly pathogenic MERS-CoV and SARS-CoV. Res. Sq..

[41] Li R., Hou Y., Huang J., Pan W., Ma Q., Shi Y., Li C., Zhao J., Jia Z., Jiang H. (2020). Lianhuaqingwen exerts anti-viral and anti-inflammatory activity against novel coronavirus (SARS-CoV-2). Pharmacol. Res..

[42] Aoki-Utsubo C., Chen M., Hotta H. (2018). Time-of-addition and temperature-shift assays to determine particular step(s) in the viral life cycle that is blocked by antiviral substance(s). Bio-Protocol.

[43] Syed G.H., Siddiqui A. (2011). Effects of hypolipidemic agent nordihydroguaiaretic acid on lipid droplets and hepatitis C virus. Hepatology.

[44] Dias S.S.G., Soares V.C., Ferreira A.C., Sacramento C.Q., Fintelman-Rodrigues N., Temerozo J.R., Teixeira L., Nunes da Silva M.A., Barreto E., Mattos M. (2020). Lipid droplets fuel SARS-CoV-2 replication and production of inflammatory mediators. PLoS Pathog..

[45] Nardacci R., Colavita F., Castilletti C., Lapa D., Matusali G., Meschi S., Del Nonno F., Colombo D., Capobianchi M.R., Zumla A. (2021). Evidences for lipid involvement in SARS-CoV-2 cytopathogenesis. Cell Death Dis..

[46] Wang M., Cao R., Zhang L., Yang X., Liu J., Xu M., Shi Z., Hu Z., Zhong W., Xiao G. (2020). Remdesivir and chloroquine effectively inhibit the recently emerged novel coronavirus (2019-nCoV) in vitro. Cell Res..

[47] Moghadasi S.A., Heilmann E., Khalil A.M., Nnabuife C., Kearns F.L., Ye C., Moraes S.N., Costacurta F., Esler M.A., Aihara H. (2023). Transmissible SARS-CoV-2 variants with resistance to clinical protease inhibitors. Sci. Adv..

[48] Zhou Y., Gammeltoft K.A., Ryberg L.A., Pham L.V., Fahnoe U., Binderup A., Hernandez C.R.D., Offersgaard A., Fernandez-Antunez C., Peters G.H.J. (2022). Nirmatrelvir resistant SARS-CoV-2 variants with high fitness in vitro. bioRxiv.

